# PROTOCOL: Effectiveness of interventions to manage acute malnutrition in children under five years of age in low‐ and middle‐income countries: a systematic review

**DOI:** 10.1002/CL2.193

**Published:** 2018-11-01

**Authors:** Jai K Das, Rehana A Salam, Marwah Saeed, Hasana Bilal, Zulfiqar A Bhutta

## Background

### The problem, condition or issue

Childhood undernutrition includes wasting (weight‐for‐height z‐score (WHZ) < ‐2SD), stunting (height‐for‐age z‐score (HAZ) < ‐2SD), underweight (weight‐for‐age z‐score (WAZ) < ‐2SD) and micronutrient deficiencies or insufficiencies (WHO 2017a). The current World Health Organization (WHO) guidelines subsume these entities into the blanket term of childhood malnutrition which is broadly categorized into acute and chronic malnutrition. Acute malnutrition is further classified on the basis of severity into moderate acute malnutrition (MAM) (WHZ between ‐3 and ‐2) and severe acute malnutrition (SAM) (WHZ < ‐3 and mid‐upper arm circumference (MUAC) < 115 mm) whereas chronic malnutrition occurs due to long‐term insufficient intake of nutrients and a complex interplay of intergenerational and environmental factors and results in stunting (UNICEF 2009). In 2017, an estimated 155 million children under five years of age were stunted and 52 million were wasted (Development Initiatives 2017). Around 45% of death among children under five years of age is associated with undernutrition (WHO 2017a). Asia and Africa still share the greatest burden of malnutrition with more than half of all stunted children and two third of all wasted children under five years of age living in Asia and over one third stunted children and a quarter of wasted children living in Africa (UNICEF 2017).

Childhood malnutrition is a major public health concern since it is associated with significant morbidity and mortality (WHO 2017). The consequences of malnutrition among infants and children can be short‐term like morbidity, mortality and disability or long‐term including impaired cognitive development, increased risk of disease due to either concurrent infections or metabolic disorders and sub optimal economic productivity (Black 2013). Undernutrition, including stunting, severe wasting, deficiencies of vitamin A and zinc, and sub‐optimum breastfeeding, has been an underlying cause of approximately one‐third of the mortality among children under five years of age (De Onis 2012; Black 2013). Childhood malnutrition is a result of a complex interplay of nutrition–specific and nutrition‐sensitive factors. Nutrition‐specific factors include inadequate food and nutrient intake, poor feeding, care giving and parenting practices, and burden of infectious diseases while nutrition‐sensitive factors include food insecurity; inadequate care giving resources at the maternal, household and community levels and limited access to health services and unhygienic environment (Bhutta 2013). Improving childhood malnutrition requires effective implementation of nutrition‐sensitive as well as nutrition‐specific interventions (Ruel 2013).

### The intervention

The existing WHO guidelines for the management malnutrition among children suggests the following (WHO 2013):

1. Early identification of children with SAM in the community through active community screening by trained community health workers and community members. Community health workers should measure the MUAC of infants and children under five years of age and examine them for bilateral pitting oedema.
2. Assessment of nutrition status in primary health‐care facilities and hospitals through routine health facility screening. Health‐care workers should assess the MUAC or the WHZ status of infants and children under five years of age and also examine them for bilateral oedema.
3. Children who are identified as having SAM should first be assessed with a full clinical examination to confirm whether they have medical complications and whether they have an appetite. Children who have appetite and are clinically well and alert should be treated as outpatients and can be managed with ready‐to‐use therapeutic food (RUTF) in amounts adjusted to their weight, to provide recommended energy intakes for recovery.
4. Children with uncomplicated SAM, not requiring to be admitted and who are managed as outpatients, should be given a course of oral antibiotics such as amoxicillin while children who are undernourished but who do not have SAM should not routinely receive antibiotics unless they show signs of clinical infection. Children admitted with SAM and with no apparent signs of infection and no complications should be given an oral antibiotic.
5. Children who have medical complications, severe oedema (+++), or poor appetite, or present with one or more Integrated Management of Childhood Illness danger signs should be treated as inpatients. Children admitted with SAM and complications such as septic shock, hypoglycaemia, hypothermia, skin infections, or respiratory or urinary tract infections, or who appear lethargic or sickly, should be given parenteral antibiotics. Children with SAM who are admitted to hospital can be transferred to outpatient care when their medical complications, including oedema, are resolving and they have good appetite, and are clinically well and alert. The decision to transfer children from inpatient to outpatient care should be determined by their clinical condition and not on the basis of specific anthropometric outcomes. Children with SAM who are discharged from treatment programmes should be periodically monitored to avoid a relapse.
6. F‐75 and F‐100 are formula diets used for the management of children with SAM in inpatient care. F‐75 (75 kcal or 315 kJ/100 mL) is used during the initial phase of treatment, while F‐100 (100 kcal or 420kJ/100 mL) is used during the rehabilitation phase. Children with SAM cannot tolerate high amounts of protein and fat and hence they are supplemented with F‐75 initially; as soon as the child is stabilized on F‐75, F‐100 is used as a “catch‐up” formula. Children with SAM who present with either acute or persistent diarrhoea, can be given RUTF in the same way as children without diarrhoea, whether they are being managed as inpatients or outpatients.
7. Children with SAM should receive the daily recommended nutrient intake of vitamin A throughout the treatment period. Children with SAM should be provided with about 5000 IU vitamin A daily, either as an integral part of therapeutic foods or as part of a multi‐micronutrient formulation.

According to these guidelines, children with complicated SAM are managed as inpatients in three phases; stabilization phase which includes fluid management for severe dehydration, correction of hypothermia, hypoglycaemia and micronutrient deficiencies and the use of antibiotics for complications; rehabilitation phase which includes increased nutrient and energy intake through therapeutic or fortified foods as well continued electrolyte and micronutrient management. Following recovery, caregivers are given appropriate nutritional training to avoid similar recurrences and instructed on the importance of sensory stimulation in children for continued emotional and physical development (Ashworth 2003). SAM among children under six months of age is increasingly being associated with higher mortality than in older infants and children (WHO 2013). The WHO guideline suggests that in infants who are under six months of age with SAM should receive the same general medical care as infants with SAM who are six months of age or older with increased focus on establishing, or re‐establishing, effective exclusive breastfeeding by the mother or other caregiver (WHO 2013).

In this review, we will assess the effectiveness of various community‐based and facility based strategies to identify and manage MAM and SAM; including the community based screening, identification management of SAM and MAM, relative effectiveness of RUTF for SAM and RUSF for MAM, effectiveness of prophylactic use of antibiotic to manage uncomplicated SAM and the effectiveness of vitamin A supplementation to manage children with acute malnutrition.

### How the intervention might work

Childhood malnutrition results in long‐term disability through cognitive impairment, delayed motor growth, poor physical performance, low‐birth weight of future offspring, behavioural issues and poor academic performance as well as sub‐optimal productivity in adulthood (Black 2008). The Community Based Management of Malnutrition (CMAM) approach has been introduced for screening and early identification of children with malnutrition to provide timely access to quality care. It enables community volunteers to identify and initiate treatment for children with acute malnutrition before they become seriously ill at home by using RUTF and routine medical care (Ashworth 2006). The CMAM approach comprises of four components: (1) community outreach and mobilization; (2) outpatient management of SAM without medical complications; (3) inpatient management of SAM with medical complications; and (4) services or programs to manage MAM, such a supplementary feeding program (Collins 2006). Early identification of children with SAM in the community is the key to prevent complications related to malnutrition and works through early case finding, referral to the management program and effective follow‐up measures. This requires contextually sensitive approaches through community assessment and mobilization (Park 2012).

Undernutrition (including all degrees of stunting, wasting, underweight and micronutrient deficiencies) has been associated with infectious diseases and children with SAM may be more susceptible to infection (Black 2013; Black 2003; Salam 2015). Current WHO guidelines suggest that prophylactic administration of antibiotics to children with uncomplicated SAM should be used to treat underlying infections; however the evidence on the current antibiotic recommendation is weak and inconclusive and requires further research considering the side‐effects, costs, and risks associated with antibiotic administration (Alcoba 2013; Picot 2012).

Supplementary feeding is expected to prevent further deterioration of nutritional status in moderately malnourished children and to restore growth and promote physiological recovery by minimizing the nutritional and energy gap (Karakochuk 2012). Supplementary foods are considered an effective strategy in the treatment and management of malnutrition either at home, facility or rehabilitation centre (Visser 2013). Supplementation promotes recovery by increasing nutrient absorption, thus improving growth and promoting development especially in the first 1000 days of life which is critical to cognitive function (Imdad 2011). A possible adverse effect of supplementary feeding interventions may be excessive and quick weight gain. Studies suggest that rapid weight correction in early childhood to reverse malnutrition can be associated with increased risk of obesity and potentially increased risk of diabetes in adulthood (Adair 2013; Norris 2011).

Micronutrient deficiencies also coexist among malnourished children and supplementation of vital micronutrients including vitamin A and zinc is required to ensure sufficiency and bioavailability within the body (Dairo 2009, Mannar 2004). Vitamin A and zinc deficiency weakens the immune system of acutely malnourished children and facilitates bacterial invasion thereby increasing the risk of morbidity and mortality due to infectious diseases (Bourke 2016; Jones 2014; Bhutta 1999; Manary 2012; Bailey 2015). However, supplementation should consider the safe upper intake levels and potential toxicology of the specific micronutrient (Renwick 2006).

### Why it is important to do this review

Despite the outlined interventions to manage childhood malnutrition (WHO 2013), there is uncertainty around the most effective methods to treat malnutrition in young children (Picot 2012). The existing WHO guidelines for the management of malnutrition also highlighted a few priority issues and research gaps (WHO 2013) that include:

1. Assessing the strategies to improve active community screening and routine health‐facility screening, and investigating barriers to service access and uptake, to enhance treatment coverage.
2. Assessing the clinical effect and cost effectiveness of giving oral antibiotics to children and infants with SAM who do not require inpatient management in non‐HIV settings.
3. Assessing the adverse effects of giving broad‐spectrum antibiotics to infants and children with SAM without complications.
4. Assessing the efficacy and effectiveness of different ready to use supplementary food (RUSF) and RUTF that comply with WHO specifications and are made from different ingredients in different regions of the world (using commercially produced RUTF as the comparison) and the comparative effectiveness of RUTF, RUSF and F‐100 for recovery of children with MAM and SAM.
5. Assessing the efficacy of daily low‐dose vitamin A supplementation compared to single high‐dose vitamin A in the treatment of children with SAM and the most effective way to improve and sustain the vitamin A status of children with SAM after discharge from treatment.

The above research gaps from the WHO guidelines have not been the topic of a comprehensive systematic review. However, there are a few existing reviews evaluating some interventions separately. Lenters 2013 undertook a systematic review to evaluate the effectiveness of approaches to managing MAM and SAM according to the WHO protocol, but the results were unclear due to lack of robust trials. Moreover there are issues related to lack of rigorous estimates due to poor adjustment for confounding variables in observational studies; heterogeneity in participants, recruitment, interventions, settings and units of measurement of outcomes (Lenters 2013). Existing reviews on management of acute malnutrition are either focused on specific population groups; specific interventions (prophylactic use of antibiotics, intravenous fluid for shock, treatment of diarrhoea, micronutrients deficiencies etc.); or there is discrepancy in the definition of undernutrition and types of therapeutic or supplementary foods ((Picot 2012; Gera 2010; Schoonees 2013; Alcoba 2013, Lazzerini 2011)). Moreover, supplementary feeding has been the topic of two reviews (Kristjansson 2015, Visser 2013) and the effectiveness of vitamin A supplementation for the treatment of SAM has also been reviewed (Manary 2012). But there is a need to comprehensively review the evidence for the management of SAM and MAM according to the current WHO protocol using facility‐ and community‐based approaches as well as the effectiveness of RUTF, RUSF, prophylactic antibiotic use and vitamin A supplementation. Therefore, the aim of this systematic review is to analyse and update the evidence on the effectiveness of recommended interventions and to assess the program and/or guidelines that have been adapted to manage children with acute malnutrition to provide a comprehensive and updated review.

## Objectives

The objectives of this review are as follows:

1. To evaluate the effectiveness of community‐based strategies such as community‐based mobilization, screening, follow‐up, counselling and education to improve screening, identification and management of SAM and MAM.
2. To evaluate the effectiveness of facility‐based strategies such as facility based screening, management and periodic follow‐up to improve screening and management of SAM and MAM.
3. To evaluate the effectiveness and relative effectiveness of various RUTF and RUSF for the management of SAM and MAM.
4. To evaluate the effectiveness of prophylactic use of antibiotic to manage uncomplicated SAM.
5. To evaluate the effectiveness of various doses of vitamin A supplements to manage children with SAM and MAM.

## Methods

### Criteria for considering studies for this review

#### Types of studies

We will include the following study designs that allow for causal inference. We will include study designs other than RCTs to include large‐scale programme evaluations, which assess the efficacy and/or effectiveness of interventions:

We will include the following study designs:

► Randomized controlled trials (RCTs), where participants were randomly assigned, individually or in clusters, to intervention and comparison groups. Cross‐over designs will be eligible for inclusion.
► Quasi‐experimental designs, which include:
  ▷ Natural experiments: studies where non‐random assignment is determined by factors that are out of the control of the investigator. One common type includes allocation based on exogenous geographical variation.
  ▷ Controlled before‐after studies (CBA), in which measures were taken of an experimental group and a comparable control group both before and after the intervention. We also require that appropriate methods were used to control for confounding, such as statistical matching (e.g., propensity score matching, or covariate matching) or regression adjustment (e.g., difference‐in‐differences, instrumental variables).
  ▷ Regression discontinuity designs; here, allocation to intervention/control is based upon a cut‐off score.
  ▷ Interrupted time series (ITS) studies, in which outcomes were measured in the intervention group at least three time points before the intervention and after the intervention.

#### Types of participants

We will include studies targeting children under five years of age with MAM and SAM in low‐ and middle‐income countries. Studies including both eligible and non‐eligible participants will only be included if the results for the eligible participant subgroup is separately provided in the study. We will use the following definition of MAM and SAM by WHO (WHO 2013):

► SAM: weight for‐ height z‐score (WHZ) < ‐3 SD, weight‐for‐height (WFH) < 70% of the median National Center for Health Statistics (NCHS) or WHO reference or mid‐upper arm circumference (MUAC) < 115mm or oedema. Complicated SAM: SAM cases without appetite and/or with medical complications. Uncomplicated SAM: SAM children with successful standard appetite test, without fever, clinical infections, or complications.
► MAM: weight‐for‐height z‐score (WHZ) between ‐2 and ‐3 standard deviations (SD), WFH equal to 70‐80% of the NCHS or WHO reference median or mid‐upper arm circumference (MUAC) of 115‐125mm.

We will exclude studies conducted on HIV populations specifically.

#### Types of interventions

The following interventions will be considered and compared against the suggested comparison groups separately:

► Community based strategies to screen, identify and manage SAM and MAM compared to standard care (e.g. active community based surveillance by community health workers (CHWs) versus no active surveillance; training of CHWs for community based screening versus no training; community based management with RUTF versus standard care practices).
► Facility based strategies to screen and manage uncomplicated SAM according to the WHO protocol compared to other standards of care (e.g. treatment for uncomplicated SAM in health facilities alone versus by CHWs and health facilities; training of health facility staff to diagnose and treat uncomplicated SAM versus no training; facility based management of SAM according to the WHO protocol versus other/locally adapted protocols).
► Community based management of children with uncomplicated SAM as outpatients with RUTF compared to standard diet, fortified blended flours (FBFs) or other locally produced foods
► RUSF for MAM compared to standard diet, or FBF or other locally produced foods.
► Prophylactic use of antibiotics in children with uncomplicated SAM compared to no antibiotics.
► Vitamin A supplementation in the management of SAM and MAM with various doses and frequency of administration.

#### Types of outcome measures

We will not use the outcomes listed below as criteria for including studies but rather as a list of the outcomes of interest. We will use denominators for the outcomes according to the intention to treat analysis to avoid misleading results. We will subgroup outcomes reported at different time points as specified under the ‘Subgroup analysis and investigation of heterogeneity’.

**Primary outcomes**

► Recovery rate (measured as the number of malnourished children recovered divided by the total number of malnourished children).
► Weight gain (measured as grams/kg/day).
► Relapse (measured as the proportion of children who re‐enrolled after they had recovered).
► Mortality (measured as the proportion of children dying under five years of age, expressed per 1,000 live births).
► Case fatality rates (measured as proportion of malnourished children dying divided by the total malnourished children).

**Secondary outcomes**

► Height gain
► MUAC gain
► Time to recover (measured as length of time between admission and discharge).
► Stunting (defined as below minus two standard deviations from median height for age of reference population).
► Wasting (defined as below minus two standard deviations from median weight for height of reference population).
► Underweight (defined as below minus two standard deviations from median weight for age of reference population).
► Infection incidence (bacteraemia, sepsis, pneumonia, urinary tract infections, meningitis, and diarrhoea).
► Adverse effects (such as side effects associated with antibiotics, drug resistance, rapid weight gain, micronutrient toxicity, etc.).
► Costs and cost effectiveness

#### Duration of follow‐up

We will attempt to standardise the effect sizes from the included studies and report the outcomes at the longest follow‐up reported.

#### Type of settings

We will include studies conducted in community or facility based settings in low and middle income countries as defined by the World Bank criteria.

### Search methods for identification of studies

#### Electronic searches

We will search the following databases: Cochrane Database of Systematic Reviews (CDSR) and the Cochrane Central Register of Controlled Trials (CENTRAL) in the Cochrane Library; World Health Organization regional databases; The Campbell Library; MEDLINE (PubMed); Embase; CINAHL; Web of Science; POPLINE; CAB abstracts and Global Health; PAHO; IndMED (indmed.nic.in/indmed.html and WHO Global Health Index. We will also search the WHO International Clinical Trials Registry Platform (ICTRP; http://www.who.int/ictrp/en/); ClinicalTrials.gov and Epistemonikos (https://www.epistemonikos.org)/. We will not restrict our searches by date, language or publication status.

#### Searching other resources

We will contact experts in relevant fields for identification of eligible studies for inclusion. We will also go through the references of identified studies and relevant reviews. We will also run citation searches of included studies in Google Scholar and Web of Sciences for other potentially relevant papers.

### Data collection and analysis

#### Description of methods used in primary research

We will include RCTs and quasi‐experimental studies. We will also include study designs other than RCTs (CBA and ITS) to include data from large‐scale programme evaluations, which assess the efficacy and/or effectiveness of interventions. One potentially eligible study is Dubrey 2008 which is a randomised, unblinded, superiority‐controlled trial. The study setting is a therapeutic feeding centre in Khartoum, Sudan. The study participants included children aged 6–59 months with SAM. The study compared once daily intramuscular injection administration with ceftriaxone for two days with oral amoxicillin twice daily for five days among children with SAM. The outcomes reported by the trial included weight gain, SAM recovery rate and case fatality ratio between the two groups.

Another potentially eligible study is Hossain 2009 which is a quasi‐experimental study. This study compared the effectiveness of locally adapted Institute of Child and Mother Health (ICMH) protocol with the WHO protocol for the management of severely malnourished children in Bangladesh. This was a non‐randomised study and the study participants were 60 severely malnourished children aged two to 59 months of age with WHZ < 70%. Children in the one group were treated with the WHO protocol while the other group was treated using the local facility protocol. The outcomes of interest included clinical improvement, weight gain, time taken to achieve target weight gain, and mortality.

#### Criteria for determination of independent findings

Before initiating the synthesis (detailed below), we will ensure that all articles reporting on the same study are appropriately linked. To ensure independence and appropriate combination of outcome constructs, syntheses will be conducted according to the type of interventions specified above. If multi‐arm studies are included, intervention groups will be combined or separated into different forest plots, and we will ensure that there is no double counting of participants. If an outcome is reported in several different metrics, we will perform unit conversions in order to pool the data. We do anticipate differences in the types of literature and we will ensure that any analysis will take possible sources of dependency into account by grouping papers into studies and ensuring that no double counting of evidence takes place when synthesizing across studies.

#### Selection of studies

Two reviewers will independently assess relevant studies by screening the titles and abstracts for inclusion. The selected studies will undergo full text evaluation and will be added based on predefined eligibility criteria. Disagreements about appropriateness of the inclusion of studies will be resolved by discussion between all review authors. Studies that meet the inclusion criteria on full text screening but upon further investigation become ineligible will appear in a ‘characteristics of excluded studies' table, along with the reasons for their exclusion. We will also attempt to contact the study authors regarding eligibility for studies where eligibility is unclear.

#### Data extraction and management

Two review authors will independently extract data on a predefined and pre‐tested data extraction sheet. We will extract the following information, where available, from relevant studies and any discrepancies will be resolved by group discussion.

**Study Method:**

► Study dates
► Location (country, urban/rural)
► Study design
► Method of recruitment
► Study context and settings

**Participants:**

► Sample size
► Age
► Gender
► Socioeconomic status
► Inclusion and exclusion criteria

**Intervention:**

► Micronutrients and vitamin A supplementation (doses and timing)
► Antibiotics (type and doses)
► Community based screening and management of malnutrition (as outpatients either at home by a health care worker, or in a community day‐care centre, residential nutrition centre or at a primary health clinic)
► Facility based screening and management of malnutrition
► Type of RUTF
► Type of supplementary feeding

**Comparison group:**

► No intervention or placebo or standard practice or other treatment.
► Type of supplementary food (RUTF, RUSF, fortified blended foods, other)

**Outcomes**

► Primary and secondary outcomes, as outlined in the types of outcome measure section. We will use denominators for the outcomes according to the intention to treat analysis to avoid misleading results

**Quality Assessment**

► On all Cochrane ‘Risk of bias’ assessment tool indicators

#### Assessment of risk of bias in included studies

Two review authors will independently assess methodological quality of studies using and any disagreements will be resolved by discussion among all review authors. The Cochrane ‘Risk of bias’ assessment tool (Higgins 2011) will be used for RCTs. We will rate each of the following components as either ‘low risk’, ‘high risk’ or ‘unclear risk’ and provide justifications for the judgements:

1. Selection bias (due to inadequate generation of a randomised sequence or concealment of allocations prior to assignment)
2. Performance bias (blinding of participants and personnel assessment)
3. Detection bias (blinding of outcome assessment)
4. Attrition bias (incomplete outcome data)
5. Reporting bias (selective reporting)
6. Other bias

For non‐randomised studies, we will use the Cochrane Effective Practice and Organisation of Care (EPOC) guidelines based on the following criteria (EPOC 2017). We will rate each of the following components as either ‘low risk’, ‘high risk’ or ‘unclear risk’ and provide justifications for the judgements:

1. Baseline outcome measurements similar
2. Baseline characteristics similar
3. Incomplete outcome data
4. Knowledge of the allocated interventions adequately prevented during study (refers to blinding of participants and personnel and blinding of outcome assessment)
5. Protection against contamination
6. Selective outcome reporting
7. Other risks of bias (e.g. bias in measurement: validity and reliability of the measures used)

For interrupted time series studies, the following criteria from EPOC will be considered (EPOC 2017). We will rate each of the following components as either ‘low risk’, ‘high risk’ or ‘unclear risk’ and provide justifications for the judgements:

1. Intervention independent of other changes
2. Shape of intervention effect pre‐specified
3. Intervention unlikely to affect data collection
4. Knowledge of the allocated interventions adequately prevented during study (refers to the blinding of outcome assessment)
5. Incomplete outcome data
6. Selective outcome reporting
7. Other risks of bias (e.g. bias in measurement: validity and reliability of the measures used; duration of observation and use of appropriate statistical modelling technique)

#### Measures of treatment effect

We will separately analyse the dichotomous and continuous outcomes. For dichotomous outcomes, we will present the results as summary risk ratios (RRs) with 95% confidence intervals (CI). We will combine incidence data as risk ratios (events per child) and rate ratios (events per child year) because of their similar interpretation and scale. We will present continuous outcome data as either a mean difference (MD), if outcomes have been measured on the same scale, or a standardized mean difference (SMD), if outcomes have been measured on different scales, with 95% CI. If outcomes are reported at multiple time points in the included studies, we will report the outcomes at the last reported time period, unless other time point points are relevant for the subgroup analysis. For studies reporting outcomes at multiple time points, we will report the last outcome reported at last follow‐up. We will conduct subgroup analysis for outcomes reported at different time periods.

#### Unit of analysis issues

We will conduct separate meta‐analysis for different study designs and for subcategories of interventions and outcomes. For cluster RCTs, if we will contact trial authors for an estimate of the intra‐cluster correlation coefficient (ICC) if the clustering effect is not accounted for in the analysis, if we are unable to contact the trial author we will then calculate an interclass correlation coefficient based on the other studies in the review and use the variance inflation factor to adjust the standard errors appropriately. Subsequently, effect sizes and standard errors will be meta‐analysed by using the generic inverse method in RevMan 5 (RevMan 2014) If there are multiple papers that describe the same trial, these will be combined and coded as a single study. For trials that include multiple intervention arms, we will select one pair (intervention and control) that satisfy the inclusion criteria of the review and exclude the rest. If > 2 intervention groups meet the eligibility criteria, then these groups will be combined into a single pair‐wise comparison group and data will be disaggregated into corresponding subgroups, or these arms will be separated into different forest plots to ensure that there is no double counting of participants. Multiple outcome estimates within the same study will be analysed separately.

#### Dealing with missing data

We will report the missing data or dropouts along with the reasons. We will contact the study authors if the missing data is not accounted for or the reasons for dropping out are unclear. If authors have accounted for missing data (i.e. multiple imputations), we will use the adjusted data within our analysis.

#### Assessment of heterogeneity

Statistical heterogeneity will be assessed using Tau^2^, I^2^ and significance of the Chi‐square test; we will also assess heterogeneity visually using forest plots. Based on prior theory and clinical knowledge, we expect clinical and methodological heterogeneity in effect sizes in this literature. Therefore, we will attempt to explain any observed statistical heterogeneity using subgroup analysis (see below Subgroup analysis and investigation of heterogeneity).

#### Assessment of reporting bias

If the number of studies is sufficient (> 10), we will use a funnel plot to visually inspect for publication bias. In addition, we will perform Egger's test to determine funnel plot asymmetry.

#### Data synthesis

Statistical analysis will be carried out separately for each intervention using Review Manager 5.3 (RevMan 2014). Separate meta‐analyses will be conducted for each type of intervention and comparison group and study design. Where analysis has not been ideal in the original papers, we would attempt to reconstruct if the data presented allows us to. Considering the expected heterogeneity in interventions, comparisons, outcomes and settings within the included studies, we will use random‐effects meta‐analyses. Where meta‐analysis is deemed inappropriate due to substantial statistical or clinical heterogeneity between studies, the findings of the included studies will be summarized in narrative form. In cases where we include multiple groups from one study, we will combine all relevant experimental intervention groups of the study into a single group, and combine all relevant control intervention groups into a single control group or include each pair‐wise comparison separately, but with shared intervention groups divided out approximately evenly among the comparisons to avoid double counts (Higgins 2011).

We will set out the main findings of the review for the primary outcomes in ‘Summary of findings’ tables prepared via the GRADE approach (Guyatt 2008) with GRADEpro 2014. We will list the primary outcome for each comparison with estimates of relative effects along with the numbers of participants and studies contributing data for those outcomes. For each primary outcome, we shall assess the quality of the evidence using the GRADE approach, which involves consideration of within‐study risk of bias (methodological quality), directness of evidence, heterogeneity, precision of effect estimates and risk of publication bias. We will rate the quality of the body of evidence for each key outcome as ‘high’, ‘moderate’, ‘low’ or ‘very low’. Randomised trials without important limitations provide high quality evidence, while observational studies without special strengths or important limitations provide low quality evidence. Non‐randomised experimental trials (quasi‐RCTs) without important limitations also provide high quality evidence, but will automatically be downgraded for limitations in design (risk of bias), such as lack of concealment of allocation.

There are five criteria that can downgrade evidence for RCTs and quasi‐RCTs. [GRADE 2004]:

► Risk of bias in individual studies
► Indirectness of evidence
► Unexplained heterogeneity or inconsistency of results
► Imprecision of results
► High probably of publication bias

There are three criteria that can upgrade the evidence for quasi‐experimental studies with no serious methodological limitations. [GRADE 2004]:

► Large magnitude of effect
► Presence of a dose response relationships
► Effect of plausible residual confounding

#### Subgroup analysis and investigation of heterogeneity

Depending on data availability, we will conduct exploratory subgroup analyses for the following subgroups:

► Age (1‐6 months, 6‐59 months)
► Duration of intervention (short‐term (< 3 months), medium‐term (3‐6 months), and long‐term (6‐12 months))
► Various formulations of supplementary foods
► Setting (Community management, primary care management, and facility management)
► Vitamin A supplementation dosage (different doses)
► Different antibiotics
► Equity (low income and disadvantaged groups versus relatively high income groups)

We will use the Chi2 test to assess subgroup differences.

#### Sensitivity analysis

We will conduct sensitivity analysis based on the risk of bias of the included studies by restricting the analysis to studies with low risk of bias for sequence generation, allocation concealment and blinding of participants to determine whether the removal of studies with high risk of bias impacts the estimates.

#### Treatment of qualitative research

We do not plan to include qualitative research.

## Review authors

**Lead review author:** The lead author is the person who develops and co‐ordinates the review team, discusses and assigns roles for individual members of the review team, liaises with the editorial base and takes responsibility for the on‐going updates of the review.

**Table:** 

Name: Jai K Das
Title: Assistant Professor
Affiliation: Aga Khan University
Address: Stadium Road
City, State, Province or County: Karachi, Sindh.
Post code: 74800
Country: Pakistan
Phone: 21‐34864717
Email: jai.das@aku.edu
**Co‐authors:**
Name: Rehana A Salam
Title: Lecturer
Affiliation: Aga Khan University
Address: Stadium Road
City, State, Province or County: Karachi, Sindh
Post code: 74800
Country: Pakistan
Phone: 21‐34864717
Email: rehana.salam@aku.edu

Name: Marwah Saeed
Title: Student
Affiliation: Aga Khan University
Address: Stadium Road
City, State, Province or County: Karachi, Sindh
Post code: 74800
Country: Pakistan
Phone: 21‐34864378
Email: marwah.m508273@student.aku.edu

Name: Hasana Bilal
Title: Research Coordinator
Affiliation: Aga Khan University
Address: Stadium Road
City, State, Province or County: Karachi, Sindh
Post code: 74800
Country: Pakistan
Phone: 21‐34864378
Email: hasanabilal86@gmail.com

Name: Zulfiqar A Bhutta
Title: Co‐Director
Affiliation: Centre for Global Child Health, The Hospital for Sick Children
Address: 686 Bay Street suite 11.9805
City, State, Province or County: Toronto, ON
Post code: M5G A04
Country: Canada
Phone: 416‐813‐7654 ext. 328532
Email: Zulfiqar.bhutta@sickkids.ca

## Roles and responsibilities

► Content: Jai K Das, Rehana A Salam, Zulfiqar A Bhutta
► Systematic review methods: Jai K Das, Rehana A Salam, Marwah Saeed, Hasana Bilal
► Statistical analysis: Jai K Das, Rehana A Salam
► Information retrieval: Rehana A Salam, Marwah Saeed, Hasana Bilal

## Sources of support

Funding for this review came from a grant from the Bill & Melinda Gates Foundation to the

Centre for Global Child Health at The Hospital for Sick Children (Grant No. OPP1137750)

## Declarations of interest

The authors are not aware of any conflicts of interest arising from financial or researcher interests

## Preliminary timeframe

Approximate date for submission of the systematic review: March 2019.

## Plans for updating the review

The corresponding author, will be responsible for any forthcoming updates to the review.

## AUTHOR DECLARATION

### Authors' responsibilities

By completing this form, you accept responsibility for preparing, maintaining and updating the review in accordance with Campbell Collaboration policy. Campbell will provide as much support as possible to assist with the preparation of the review.

A draft review must be submitted to the relevant Coordinating Group within two years of protocol publication. If drafts are not submitted before the agreed deadlines, or if we are unable to contact you for an extended period, the relevant Coordinating Group has the right to de‐register the title or transfer the title to alternative authors. The Coordinating Group also has the right to de‐register or transfer the title if it does not meet the standards of the Coordinating Group and/or Campbell.

You accept responsibility for maintaining the review in light of new evidence, comments and criticisms, and other developments, and updating the review at least once every five years, or, if requested, transferring responsibility for maintaining the review to others as agreed with the Coordinating Group.

### Publication in the Campbell Library

The support of the Coordinating Group in preparing your review is conditional upon your agreement to publish the protocol, finished review, and subsequent updates in the Campbell Library. Campbell places no restrictions on publication of the findings of a Campbell systematic review in a more abbreviated form as a journal article either before or after the publication of the monograph version in Campbell Systematic Reviews. Some journals, however, have restrictions that preclude publication of findings that have been, or will be, reported elsewhere and authors considering publication in such a journal should be aware of possible conflict with publication of the monograph version in Campbell Systematic Reviews. Publication in a journal after publication or in press status in Campbell Systematic Reviews should acknowledge the Campbell version and include a citation to it. Note that systematic reviews published in Campbell Systematic Reviews and co‐registered with Cochrane may have additional requirements or restrictions for co‐publication. Review authors accept responsibility for meeting any co‐publication requirements.

**I understand the commitment required to undertake a Campbell review, and agree to publish in the Campbell Library. Signed on behalf of the authors**:

**Table:** 

**Form completed by: Jai K Das**	**Date:27 October 2018**

## Appendix

1. Search strategy

PubMed Search Strategy (searched in title, abstract and/or keyword searches)

#1. “Infant”[Mesh]

#2. “Child, Preschool”[Mesh]

#3. Infant*

#4. Toddler*

#5. Baby OR babies

#6. Newborn* OR Neonat*

#7. Preschool* OR Kindergarten* OR Under‐5s OR “Under 5s” OR “Under 5”

#8. #1 OR #2 OR #3 OR #4 OR #5 OR #6 OR #7

#9. “Severe Acute Malnutrition”[Mesh]

#10. “Infant Nutrition Disorders”[Mesh]

#11. “Nutrition Disorders”[Mesh]

#12. “Severe Acute Malnutrition” OR SAM

#13. “Moderate Acute Malnutrition” OR MAM

#14. “Protein‐Energy Malnutrition”[Mesh]

#15. Undernutrition OR under‐nutrition

#16. Malnourish*

#17. Malnutrition

#18. Stunted OR wasted OR wasting OR “Wasting Syndrome”[Mesh]

#19. Starve* OR Starvat* OR “Starvation”[Mesh]

#20. “Vitamin A” OR “Vitamin A Deficiency” “Vitamin A”[Mesh]

#21. “Iron”[Mesh] OR “Iron deficiency” OR “Fe deficiency” OR “Anemia”[Mesh]

#22. Zinc OR “Zinc deficiency OR “Zn deficiency” OR “Zinc”[Mesh]

#23. #9 OR #10 OR #11 OR #12 OR #13 OR #14 OR #15 OR #16 OR #17 OR #18 OR #19 OR #20 OR #21 OR #22

#24. “Food”[Mesh]

#25. “Infant Food”[Mesh]

#26. “Food, Fortified”[Mesh]

#27. “Food, Formulated”[Mesh]

#28. “Dietary Supplements”[Mesh]

#29. “Fortified Food*”

#30. “Diet* Supplement*”

#31. “Ready to use therapeutic food” OR RUTF

#32. “Ready to use supplementary food” OR RUSF

#33. “Ready to use food*” OR RUF

#34. F100 OR F75

#35. CTC

#36. “Vitamin A Supplement*”

#37. “Micronutrient* Supplement*”

#38. “Dietary Fats”[Mesh]

#39. “Dietary Proteins”[Mesh]

#40. FBF

#41. “Corn soy*”

#42. “Wheat soy* blend*”

#43. “Rice mild blend*”

#44. “Milk rice blend*”

#45. “Pea wheat blend*”

#46. “Cereal pulse blend*”

#47. “Lipid‐based nutrient supplement*”

#48. Nutributter

#49. “Milk Proteins”[Mesh]

#50. “Community based management of malnutrition” OR CMAM

#51. “Amoxicillin”[Mesh]

#52. “Cotrimoxazole”[Mesh]

#53. Bacteraemia*

#54. Gentamicin

#55. “Penicillin G”[Mesh]

#56. “Chloramphenicol”[Mesh]

#57. “Ceftriaxone”[Mesh]

#58. “Ciprofloxacin”[Mesh]

#59. “Inpatient management” OR “In‐patient management” OR IMCI OR IMNCI

#60. “Community based management”

#61. “Facility based management”

#62. Prophyla* AND antibiotic*

#63. #24 OR #25 OR #26 OR #27 OR #28 OR #29 OR #30 OR #31 OR #32 OR #33 OR #34 OR #35 OR #36 OR #37 OR #38 OR #39 OR #40 OR #41 OR #42 OR #43 OR #44 OR #45 OR #46 OR #47 OR #48 OR #49 OR #50 OR #51 OR #52 OR #53 OR #54 OR #55 OR #56 OR #57 OR #58 OR #59 OR #60 OR #61 OR #62

#64. “Morbidity”[Mesh]

#65. “Mortality”[Mesh]

#66. Death*

#67. Relapse*

#68. Recovery

#69. #64 OR #65 OR #66 OR #67 OR #68

#70. #8 AND #23 AND (#63 OR #69)

#71. Age Filters Applied: Infants 1‐23 months; birth‐23 months; Preschool child 2‐5 years
